# Distal femoral osteotomy for the valgus knee: indications, complications, clinical and radiological outcome

**DOI:** 10.1007/s00402-023-04923-w

**Published:** 2023-06-06

**Authors:** Petros Ismailidis, Corinna Schmid, Julika Werner, Corina Nüesch, Annegret Mündermann, Geert Pagenstert, Christian Egloff

**Affiliations:** 1 Department of Orthopaedics and Traumatology, University Hospital Basel, Spitalstrasse 21, 4031 Basel, Switzerland; 2 Department of Clinical Research, University of Basel, Schanzenstrasse 55, 4056 Basel, Switzerland; 3 Department of Spine Surgery, University Hospital Basel, Spitalstrasse 21, 4031 Basel, Switzerland; 4 Knee Institute Basel, Clarahof, Clinic of Orthopaedic Surgery, Clarahofweg 19a, 4058 Basel, Switzerland; 5 Present Address: Department of Biomedical Engineering, University of Basel, Basel, Switzerland

**Keywords:** Distal femoral osteotomy, DFO, Valgus knee, Knee osteoarthritis

## Abstract

**Introduction:**

The aim of this study was to describe the indications and technical aspects of medial closing and lateral opening distal femoral osteotomy (MCDFO and LODFO) for patients with a valgus knee and to report clinical and radiological outcomes and complications.

**Methods:**

Over 6 years, 28 DFOs (22 MCDFO, 6 LODFO) were performed in 22 Patients. In this cohort study, we retrospectively analyzed clinical and radiological outcome measures as well as complications.

**Results:**

The median (range) age was 47 (17–63) years, height 1.68 (1.56–1.98) m, body mass 80 (49–105) kg, and body mass index (BMI) 27.4 (18.6–37.0) kg/m^2^. The clinical follow-up was 21 (7–81) months, the need for total or unicompartmental knee arthroplasty (TKA/UKA) and hardware removal was followed up for 59 (7–108) months postoperatively. Preoperatively, hip-knee-ankle angle (HKA, negative values denote varus) was 7.0 (2.0–13.0)°, mechanical lateral distal femoral angle (mLDFA) was 83.7 (79.9–88.2)°, and mechanical proximal tibial angle (MPTA) was 89.0 (86.6–94.5)°. Postoperatively, HKA was −1.3 (−9.0–1.2)° and mLDFA was 90.8 (87.3–97.3)°. The incidence of minor and major complications was 25% and 14%, the incidence of delayed and nonunion was 18% and 4%, respectively. At the last follow-up, 18% of the patients had pain at rest, 25% during activities of daily living, and 39% during physical activity, and 71% were satisfied with the outcome. 7% of the cases received a TKA/UKA, 71% received a hardware removal.

**Conclusion:**

DFO is a reasonable treatment for lateral osteoarthritis in younger patients to avoid disease progression and the need for an UKA/TKA. However, there is a long rehabilitation time, a considerable risk for complications, and a high need for hardware removal. While many patients experienced symptoms at the long-term follow-up, most were satisfied with the outcome. Appropriate patient information is essential.

**Level of evidence** Level IV, Case Series.

**Trial registration number** NCT04382118, clinicaltrials.gov, May 11, 2020.

## Introduction

Valgus knee is defined as a lower extremity where the mechanical axis passes laterally of the knee joint center [16]. Valgus alignment has been shown to increase the risk of chondral damage because of abnormal joint loading, as well as the risk of early onset and progression of valgus osteoarthritis of the knee [2, 7, 9, 24]. Furthermore, valgus alignment predicts a decline in physical function in patients with knee osteoarthritis [2, 7, 9, 24]. Consequently, different corrective osteotomies around the knee have been used for the correction of valgus malalignment. The incidence of valgus alignment is lower than that of varus alignment, and valgus-correcting osteotomies have been reported far less in the literature than varus-correcting osteotomies [3, 22].

Valgus-correcting osteotomies can be performed both at the femur and at the tibia. The site of the osteotomy is dictated by the location of the deformity. In most valgus knees, the deformity is located at the femur, and accordingly distal femoral osteotomy (DFO) is the most common osteotomy for valgus knees [7]. Two different DFOs can be performed, namely the medial closing osteotomy (MCDFO) and the lateral opening distal femoral valgus osteotomy (LODFO). Both types of DFOs have been established as acceptable surgical methods to correct valgus alignment, relieve symptoms and decrease disease progression in patients with valgus osteoarthritis [25].

MCDFO and LODFO each have technical and practical advantages and disadvantages and been propagated by different surgeons. Three systematic reviews have failed to detect a superiority of one method over the other [7, 13, 31]. Furthermore, results and complications vary among studies. This is, at least partially, due to the limited amount of available literature. The reviews reported 16 to 23 studies with the number of performed DFOs varying from 7 to 40 in each study. To date, only three studies reported on both MCDFOs and LODFOs. All reviews underline the need of additional clinical data on DFOs. Therefore, the aim of this retrospective study was to describe the indications and technical aspects of MCDFO and LODFO for patients with a valgus knee and to report clinical and radiological outcomes as well as complications. The primary endpoint was the clinical and radiological outcome of DFO, and the secondary endpoint was the need for conversion to total or unicompartmental knee arthroplasty (TKA/UKA).

## Materials and methods

### Ethics

This study was approved by the regional ethics board (EKZN 2020-00108) and registered at clinicaltrials.gov (Trial Registration Number: NCT04382118).

### Study design, inclusion criteria

This was a retrospective cohort study. All patients who received a DFO in our institution between 1/2012 and 1/2018 were included in this study. Operations were performed by three consultants for orthopaedic surgery. The clinical and radiological data were collected as part of the postoperative follow-up and extracted from patient records for analysis.

### Preoperative measurements, indications for MCDFO and LODFO

Patients with lateral compartment osteoarthritis and valgus knee alignment of the knee were considered for valgus-correcting osteotomy. Presence of medial compartment osteoarthritis or inflammatory arthritis were contraindications for a valgus-correcting osteotomy. Patients older than 65 years were generally addressed with a partial or total knee arthroplasty rather than with an osteotomy. DFO or high tibial osteotomy (HTO) or a combination of both were performed to correct the valgus alignment according to the location of the deformity as described below.

**Fig. 1:**
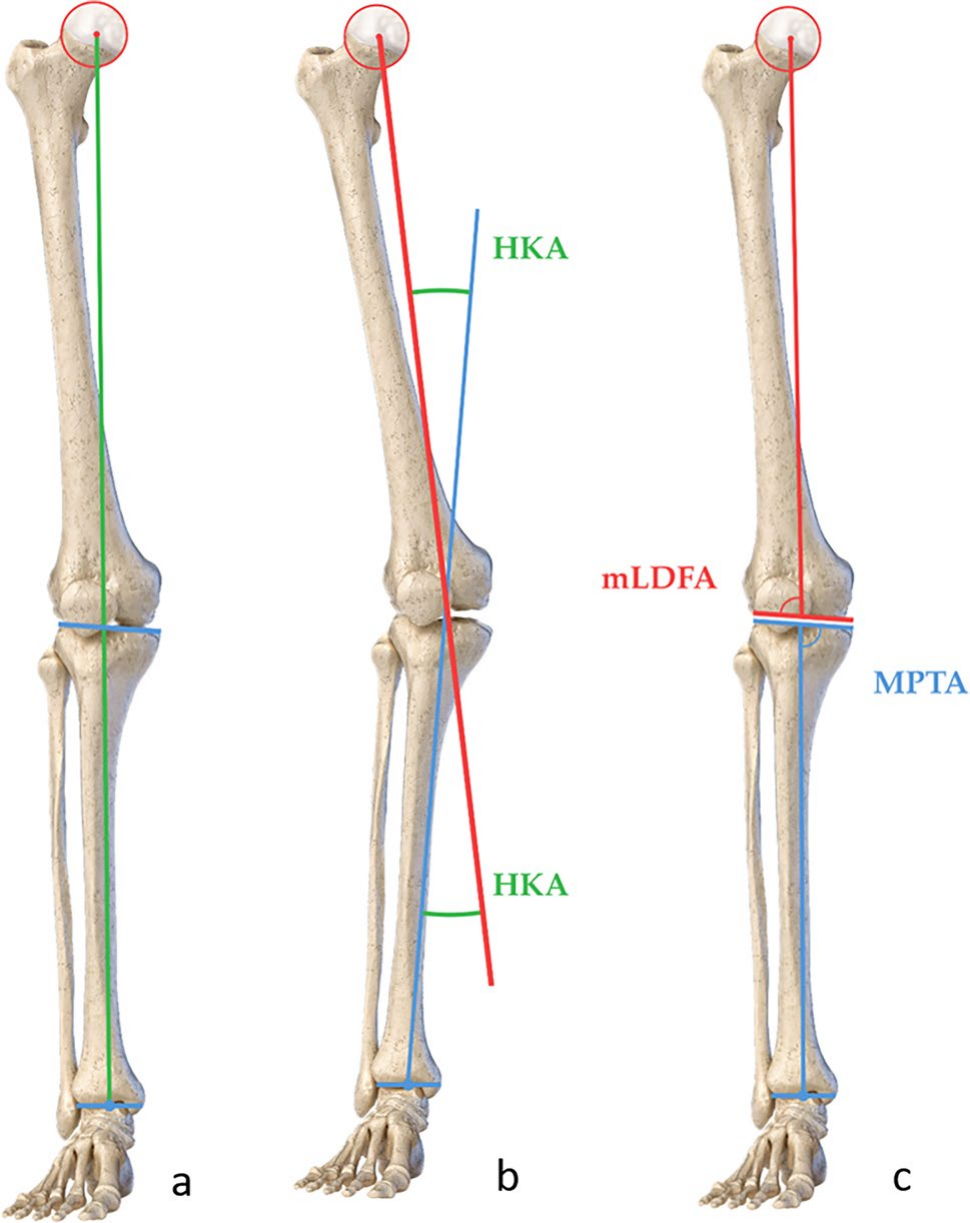
Radiological measurements performed preoperatively. **a** The mechanical axis of the leg (green line) is a line passing through the center of the hip joint (center of the femoral head, red point) and the center of the ankle joint (midpoint of tibial plafond, blue point). A mechanical axis crossing the knee lateral to the center of the knee joint, defines a valgus knee. **b** The mechanical axis of the femur (red line) is defined as a line connecting the center of the hip joint to the center of the knee (center of the tibial spines). The mechanical axis of the tibia (blue line) is defined as a line connecting the center of the knee to the center of the ankle joint. The hip knee ankle angle (HKA, marked green, positive values indicate valgus, normal values −1 ± 3°) is defined as the ankle between the mechanical axis of the femur and the mechanical axis of the knee. **c** The mechanical lateral distal femoral angle (mLDFA, < 90° indicating valgus, normal values 87.5 ± 2°) is defined as an angle between the mechanical axis of the femur and the distal femur joint line. The medial proximal tibial angle (MPTA, < 90° indicating varus, normal values 87.5 ± 2°) is defined as the ankle between the mechanical axis of the tibia and the tibial plafond

Anteroposterior (AP) long leg standing radiographs were performed preoperatively and 6–8 weeks postoperatively. Furthermore, AP and lateral X-rays of the knee were performed preoperatively, 6–8 weeks postoperatively and then every 6–12 weeks until bone union was achieved. The mechanical axis of the leg was measured according to the Mikulicz line to define the valgus malalignment [16]. Valgus alignment was defined as a mechanical axis crossing the knee lateral to its center[16]. The Hip Knee Ankle Angle (HKA, negative values denote varus, normal values −1 ± 3° [5]) was calculated as a measure of the valgus malalignment. The mechanical medial proximal tibial angle (MPTA, < 90° indicating varus, normal values 87.5 ± 2.0) and the mechanical lateral distal femoral angle (mLDFA, < 90° indicating valgus, normal values 87.5 ± 2.0) were calculated in each case [16] (Fig. 1). A symptomatic valgus knee (HKA > 0°) with a femoral based deformity (mLDFA < 87.5°, MPTA in normal range) was considered as an indication for a DFO.

Magnetic resonance imaging (MRI) of the knee was performed preoperatively in all cases for the evaluation of potential meniscal and chondral pathologies. Torsional deformities of the tibia [27] and of the femur [23] were assessed clinically and investigated with MRI and computer tomography scans only if the clinical exam indicated pathologic values.

**Fig. 2:**
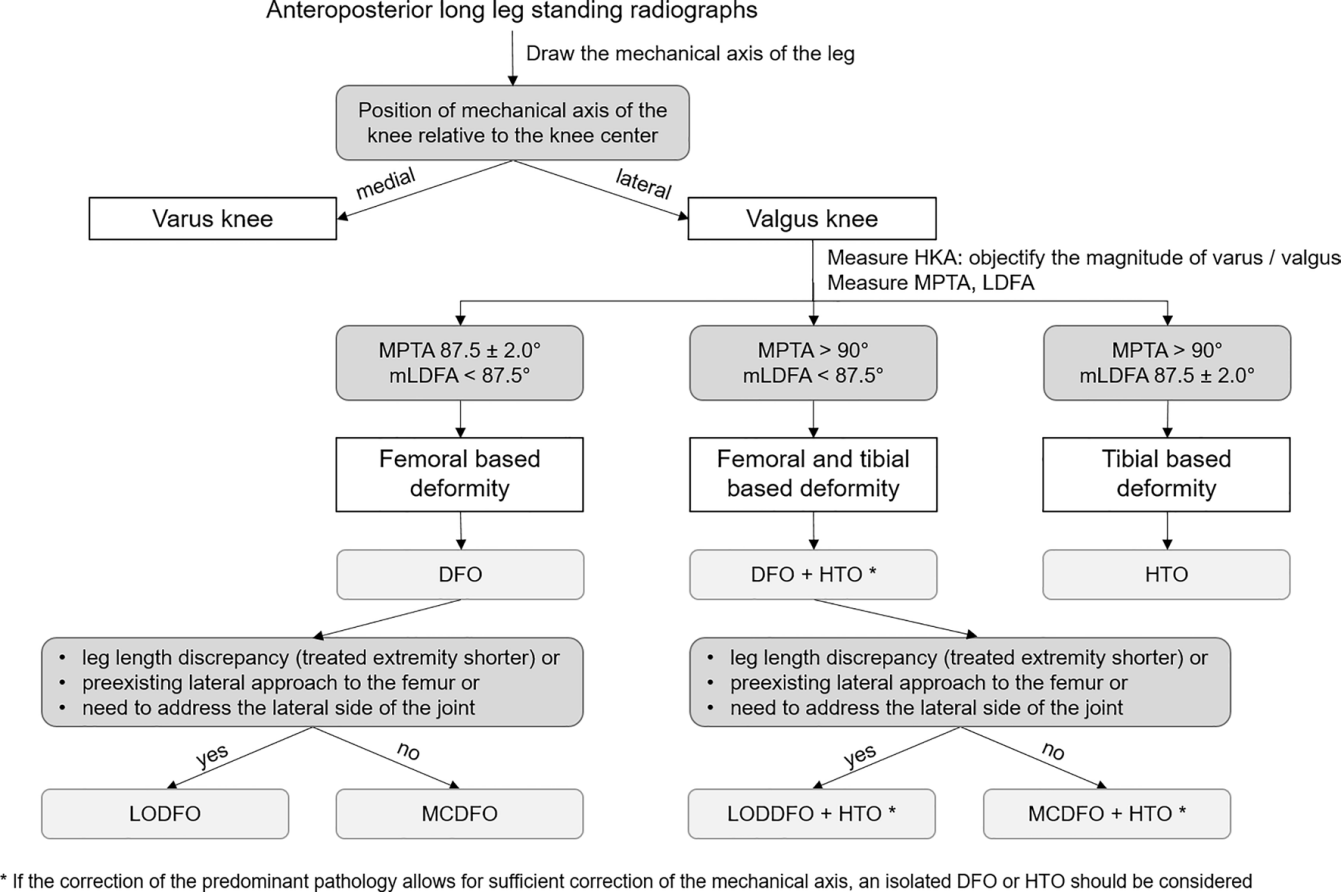
Diagnostic algorithm applied. The mechanical axis of the limb is drawn to define the valgus alignment. The HKA is measured to define the amount of valgus present. The mLDFA and the MPTA are measured to define the site of the deformity responsible for the valgus limb alignment. In case of a femoral based deformity, a MCDFO is performed except if specific indications for a LODFO are present

We applied a diagnostic and treatment algorithm to guide our decision for the choice of osteotomy (Fig. 2). MCDFO was the method of choice in absence of specific indications for LODFO because MCDFO creates direct bone apposition without a need for a bone graft resulting in greater construct stability, thus allowing early weight bearing. Furthermore, MCDFO reduces the potential risk of delayed and non-union, and facilitates access to the medial side of the knee in case of need of concomitant procedures [7, 8]. Specific indications for LODFO were leg length discrepancy with the arthritic extremity being shorter than the contralateral side (in this case performing a MCDFO would aggravate the leg length discrepancy), preexisting lateral approach to the femur (e.g., in case of posttraumatic deformity with earlier osteosynthesis of the femur performed via lateral subvastus approach), or need to address the lateral side of the joint (e.g., for a lateral patellofemoral release).

### Surgical methods: technical considerations

The TOMOFIX® Osteotomy System (Synthes GmbH, Oberdorf, Switzerland) was used in all but two cases (same patient) where a PHILOS® plate system (Synthes GmbH, Oberdorf, Switzerland) was used because of specific patient anatomy considerations making this implant more appropriate. MCDFO was performed through a medial subvastus approach, LODFO through a lateral subvastus approach. Autologous bone graft was used in only one case of LODFO. Correction was performed with the goal of neutral alignment, with an HKA of 0°. The osteotomy was performed at the metadiaphyseal region with the goal of a hinge at the upper border of the lateral respectively medial femoral condyle to minimize the risk of an unstable hinge fracture [10, 12, 15]. Postoperatively, the patients were mobilized with partial weight bearing of 15 kg for 6 weeks. No knee brace was applied. Physiotherapy was initiated at the first postoperative day, continuous passive motion and active range of motion (ROM) exercises were included. After the first follow up gradual increase of weight bearing was allowed. Additional loading and return to sports was allowed when bone union was achieved.

### Outcome measures: clinical and radiological outcome, need for hardware removal, need for conversion to TKA/UKA, complications

**Fig. 3:**
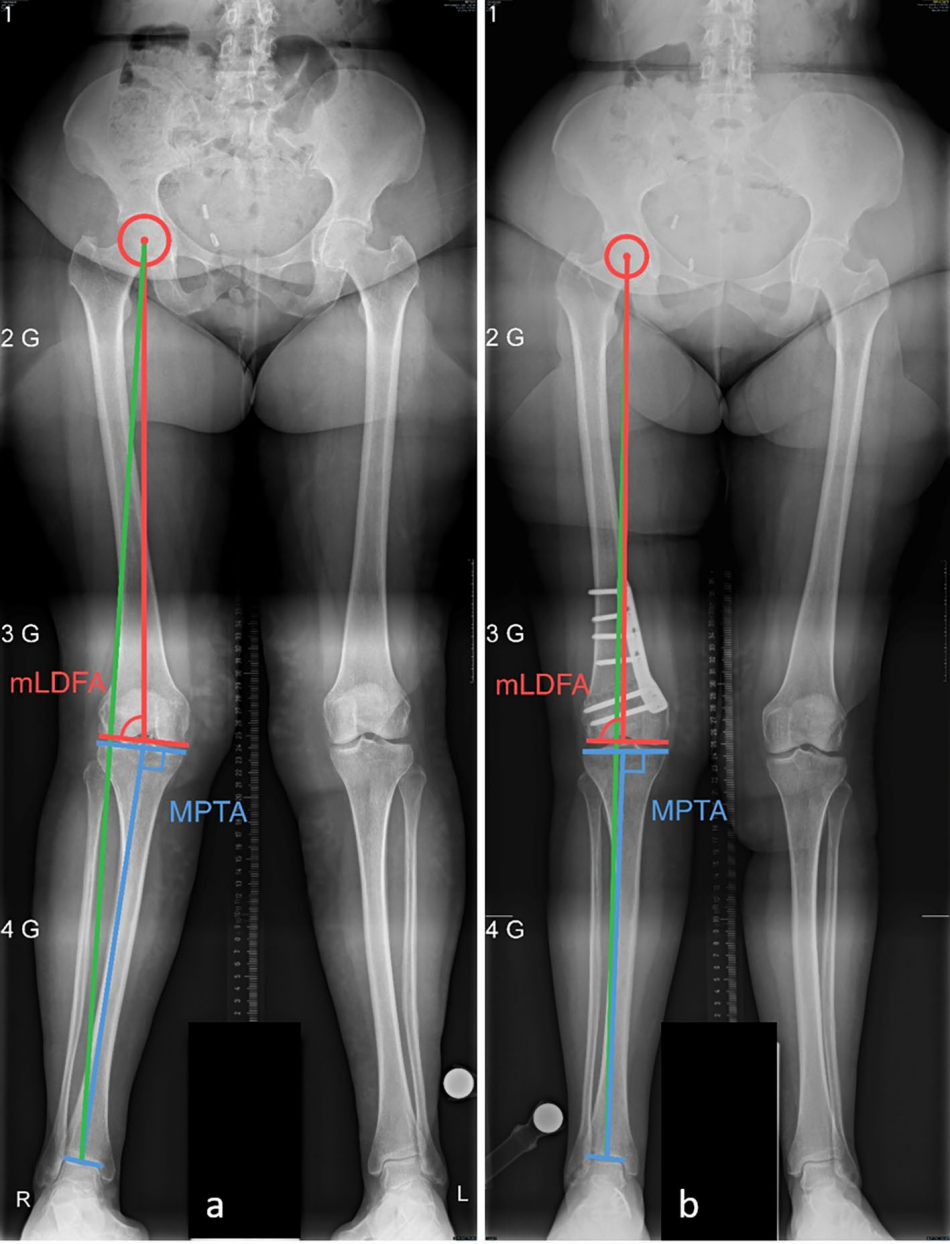
Patient receiving a MCDFO. **a** Preoperative images showing a mechanical axis (green line) crossing the knee lateral to its center, an HKA of 9°, a mLDFA of 85° and a MPTA of 90°. **b** Postoperative images showing a mechanical axis passing through the knee center with an HKA of -1°and a mLDFA of 91°

**Fig. 4:**
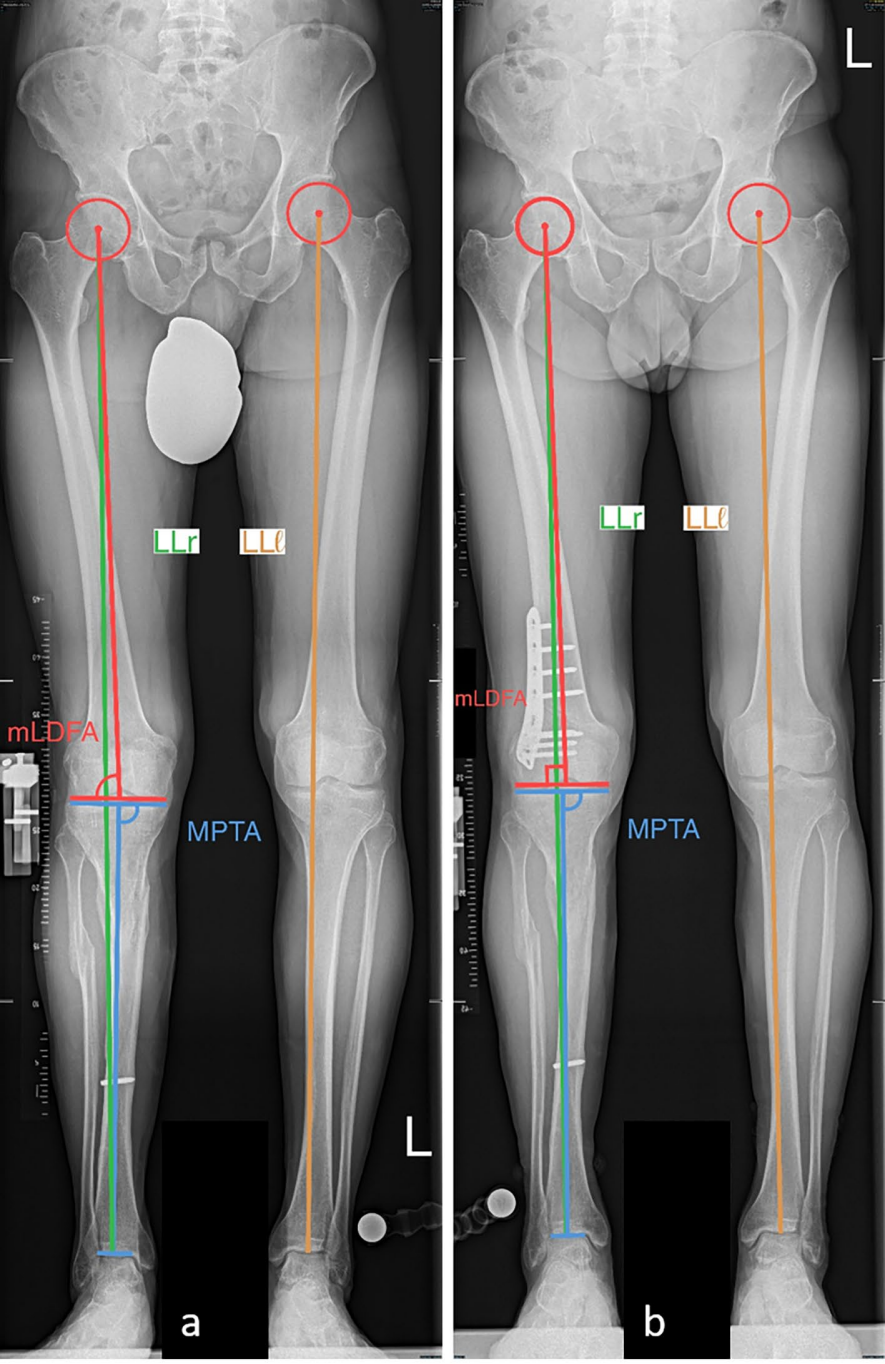
Patient receiving a LODFO. **a** Preoperative images showing a mechanical axis (green line) crossing the knee lateral to its center, an HKA of 4°, a mLDFA of 86° and aMPTA of 89°. Note the length leg discrepancy (LLD) of 2 cm, leg length right 92 cm (LLr, green line), leg length left 94 cm (LLl, orange line) which defined the indication for a LODFO. **b** postoperative images showing a mechanical axis passing through the knee center with an HKA of 1° and a mLDFA of 88°. LLD corrected to 0.5 cm, LLr 93.5 cm (green line), LLl 94 cm (orange line)

**Fig. 5:**
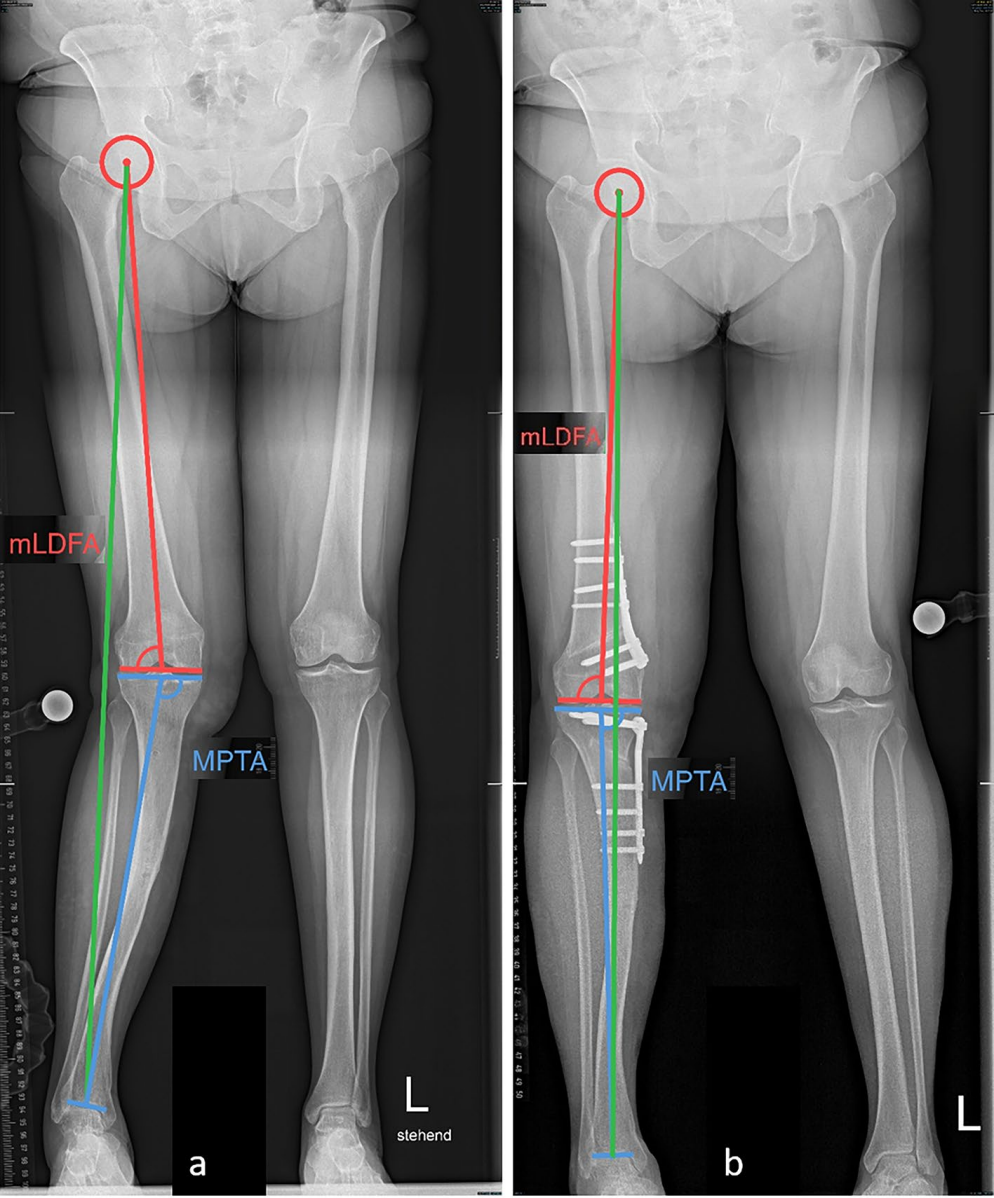
Patient receiving a MCDFO and HTO. **a** Preoperative images showing a mechanical axis (green line) crossing the knee lateral to its center, an HKA of 13°, a mLDFA of 84° and a MPTA of 94.5°. Note that since the deformity was femoral and tibial based, a MCDFO and HTO was performed. **b** Postoperative images showing a mechanical axis (green line) crossing the knee slight medial to its center an HKA of −2° a mLDFA of 90° and a MPTA of 88°

Clinical outcome measures included presence of pain at rest, during activities of daily living (ADL) and during physical activity as well as knee ROM preoperatively and at the latest follow-up. Patient satisfaction was assessed at the final follow-up. The need for hardware removal and the need for unicompartmental or total knee arthroplasty (UKA, TKA) was assessed during the follow-up period and at the point of data analysis through a follow-up call to maximize the period of documentation for these critically important outcomes. Radiological outcome measures included Kellgren–Lawrence (K/L) arthritis score [11], HKA, mLDFA before and 6 weeks after the operation as well as the MPTA preoperatively (Figs. 3, 4, 5).

Complications were documented and classified as minor (Grade I and II) or major (Grade III–V) [26]. Briefly, complications requiring no change in the routine postoperative course or only necessitating a change in outpatient management were classified as minor complications while those requiring surgical management or affecting long-term morbidity were classified as major complications. Accordingly, hardware failure, loss of correction, deep infection, vessel or nerve injury, complex regional pain syndrome, joint stiffness requiring re-operation and non-union were classified as major complications. Superficial infection, hematoma, transient nerve dysfunction or delayed union were considered minor complications [26, 28].

Bony union was assessed separately. Since there is no generally accepted definition for bony, delayed and nonunion [30] a generic definition was used for this study. Bony union was defined as a bone bridging of the osteotomy in at least three out of four cortices in the AP and lateral X-rays with absence of pain over the osteotomy site with palpation as well as with weight bearing [6]. Delayed union was defined as the absence of bone union 6 months postoperatively and nonunion as the absence of bone union 12 months postoperatively. Smoking was assessed separately since it has been associated with increased delayed and nonunion rates [17] to the extent that some authors consider smoking a contraindication to osteotomy [18, 25].

## Results

### Demographic data

Twenty-eight DFOs (22 MCDFO, six LODFO) performed in 22 Patients (six bilateral; 17 female, five male) were included in this study. One patient had a femoral and tibial based deformity and received a combined MCDFO and HTO (Fig. 5). The median (range) age was 47 (17–63) years, height 1.68 (1.56–1.98) m, body mass 80 (49–105) kg, and body mass index (BMI) 27.4 (18.6 -37) kg/m^2^. The demographic data of each group is presented in Table 1.

**Table 1:** Demorgraphic characteristics, incidence of smoking, delayed union, nonunion, hardware removal, minor and major complications as well as need for joint replacement

	All distal femoral osteotomies	Medial closing wedge distal femoral osteotomies	Lateral opening wedge distal femoral osteotomies
Number of knees/patients	28/22	22/17	6/5
Sex m/f	5/17	4/13	1/4
Age (years)	47 (17–63)	48 (17–63)	45 (31–61)
Body mass (kg)	80 (49–105)	72 (49–105)	84.0 (80–98)
Height (m)	1.68 (1.56–1.98)	1.69 (1.58–1.98)	1.65 (1.56–1.78)
BMI (kg/m^2^)	27.4 (18.6–37.0)	26.6 (18.6–37.0)	29.4 (27.1–34.7)
Smoking	25%	32%	0%
Delayed union	14%	18%	0%
Nonunion	4%	5%	0%
Hardware removal	71%	73%	67%
Major complications	14%	14%	17%
Minor complications	25%	27%	17%
Need for joint replacement	7%	9%	0%

Values are given as median (range)

### Indications

The indication for the DFO was a valgus osteoarthritis in all cases. In 71% of the cases, it was a degenerative valgus osteoarthritis and in 29% of the cases, it was a posttraumatic valgus osteoarthritis. Out of the cases having a valgus osteoarthritis 30% had additionally a patellar maltracking/patellar instability. Six LODFOs (20% of the DFOs) were performed. The indications for the LODFO were leg length discrepancy in two cases, the need for a lateral approach to perform a lateral patellofemoral release and lengthening of lateral retinaculum because of lateral patellar compression syndrome in two cases, the need of a lateral approach to protect a previous medial patellofemoral ligament reconstruction in one case and the need to correct a preexisting superficial scar tissue in one case. Intraoperative fracture of the contralateral hinge occurred in 32% (MCDFO 27%, LODFO 33%) of the cases. In 75% of the cases, concomitant procedures were performed at the time of the DFO. Out of these, the most common were lateral meniscectomy in 32%, chondroplasty/microfracturing in 18% and medial patellofemoral ligament reconstruction in 11%.

### Clinical and radiological outcome

The median (range) clinical follow-up was 21 (7–81) months.

#### Clinical outcome

At the last follow-up, 18% had pain at rest, 25% during ADL and 39% during physical activity. The median (range) flexion was 130° (80–140°), two knees had a flexion of 90° and one a flexion of 80° while the rest had flexion above 100°. All knees achieved full knee extension, four knees (two patients bilateral) had a hyperextension of 5° 0.71% of the patients were satisfied with the outcome. 21% of the patients received some pain medication at the final follow-up. 93% of the patients had returned to work at the final follow-up, while 86% could return to the occupation they had preoperatively.

#### Radiological outcome

Preoperatively, the median (range) HKA was 7.0 (2.0–13.0)°, mLDFA was 83.7 (79.9–88.2)°, and MPTA was 89.0 (86.6–94.5)°. Postoperatively, the HKA was −1.3 (−9.0–1.2)° and mLDFA was 90.8 (87.3–97.3)°. Preoperatively, five knees had a K/L Score 1, seven knees a K/L Score 2, 14 knees a K/L Score 3 and two knees a K/L Score 4. At the final follow-up, 21 knees showed no progression of the K/L Score, five knees had an increase of one point and two an increase of two points. Out of the seven knees showing osteoarthritis progression, three had received a lateral partial meniscectomy at the time of DFO (two knees showed an increase of two points and one an increase of one point).

#### Complications

The incidence of major and minor complications was 14% (MCDFO 14%, LODFO 17%) and 25% (MCDFO 27%, LODFO 17%) respectively. Each of the major complications documented occurred once, namely, nonunion, stiffness requiring mobilization under anesthesia, loss of correction, popliteal nerve-vessel injury. Regarding the minor complications, one patient presented with a transient peroneal nerve palsy, one patient with a superficial wound healing problem and five patients with a delayed union.

#### Bone union

The median (range) time to achieve bone union was 6 (2–10) months (MCDFO 5.5 (2–9) months, LODFO 6 (4–10) months). The incidence of delayed union was 14% (MCDFO 18%, LODFO no delayed union) and the incidence of nonunion 4% (MCDFO 5%, LODFO no nonunion). The patient with a nonunion was a smoker while none of the patients with delayed union were smokers. Overall, there was a 25% incidence of smoking. Four out of the five patients having a delayed union or nonunion had an intraoperative fracture of the contralateral hinge.

#### Hardware removal after DFO

Hardware removal was performed in 71% of the cases (MCDFO 73%, LODFO 67%).

#### Need for joint replacement after DFO

The need for a TKA/UKA was followed up for 59 (7–108) months postoperatively through follow up calls. 7% of the cases received a TKA/UKA, namely, one patient received a lateral UKA, and one a TKA. None of the patients who received a prosthesis were in the group of patients who received a partial meniscectomy at the time of the DFO. One patient, received a bilateral patellofemoral joint replacement, this was not considered a “need for joint replacement after DFO”, since the primary goal of DFO is not to prevent a patellofemoral joint replacement.

## Discussion

This study reports the indications as well clinical and radiological outcomes and complications of DFOs performed in our institution over a period of 6 years. The most important finding was that—although DFO was to a large extent a successful treatment for lateral osteoarthritis in young patients to avoid disease progression and the need for an early UKA/TKA—there is a long rehabilitation time, a considerable risk for complications, and a high need for hardware removal. Furthermore, many patients still experience some symptoms at the final follow-up.

### Indications of DFO, MCDFO and LODFO

Three systematic reviews failed to prove the superiority of MCDFO or LODFO for correction of valgus osteoarthritis [7, 13, 31]. Most surgeons choose one of the two methods, and perform the same method in all cases [7, 13, 31]. Consequently, out of 23 studies identified in the systematic reviews, only three reported on MCDFOs and LODFOs, while the others performed the same method (either MCDFO or LODFO) in all patients [7, 13, 31].

However, we believe that no single method serves all patients, and hence both methods are applicable with appropriate patient selection. The advantages of MCDFO include the direct bone opposition without bone graft with the potential of reducing delayed and nonunion and allowing earlier weight bearing. Therefore, MCDFO could be more appropriate for patients who have increased risk of impaired bone healing (e.g., smokers) as well as for patients who have difficulties to partially bear weight. However, MCDFO is not appropriate for patients with a leg length discrepancy, where the arthritic extremity is shorter than the contralateral side and does not allow direct access to the lateral side of the knee. The algorithm illustrated in Fig. 2 represents a way of approaching the treatment of valgus knees using both MCDFOs and LODFOs in the appropriate patient groups. It must be considered that no algorithm can cover all cases, and the choice of MCDFO or LODFO for a specific patient ultimately remains a surgeon's decision. Some patients may have characteristics qualifying for both LODFO and MCDFO. For instance, a patient might have preexisting lateral approach—theoretically dictating an LODFO—and a leg length discrepancy, with the operated leg being longer, which according to the algorithm would be an indication for a MCDFO. Furthermore, patients might present with symptomatic valgus osteoarthritis with mLDFA or HKA values marginally in the normal range, and therefore theoretically not qualifying for a DFO according to the algorithm. In such cases, it remains the surgeon's responsibility to consider the indication for a DFO and carefully weigh all factors pointing to each method.

The data of this study and the study design were not sufficient for comparing the outcomes of MCDFO and LODFO. Nevertheless, regarding the main points of consideration concerning DFO techniques—namely, complications, bone union and hardware removal rates—the two techniques had very similar results.

### Clinical outcome: setting realistic expectations

In the present study, 71% of patients receiving a DFO were satisfied with the outcome. However at the final follow-up, 25% of patients had pain during ADL and 39% during physical activity, and 21% still received some pain medication. This result indicates that—although DFO is a reasonable treatment with roughly two thirds of the patients being satisfied with the result—a completely pain free situation cannot be guaranteed and some pain may still be present mid- to long term. Patient education, setting realistic goals and expectations is essential for improving patient satisfaction. Recent literature supports these findings. Rensing et al. reported 58% of military personnel receiving a DFO never returning to duty [21]. A systematic review by Khalik et al. [1] reported an 81.8% rate of return to work and 72.8% return to preoperative working activity level after DFO. These data are likely an optimistic estimation of the overall DFO outcomes because results of only a fraction of the DFOs performed worldwide are published, originating mostly from specialized high-volume centers.

### Radiological outcome

In this cohort study we aimed at correcting the alignment to neutral. This is the goal alignment for almost all studies reporting on DFO [19] to avoid overloading of the medial compartment, even though biomechanical studies have suggested that slight overcorrection to varus could be beneficial regarding joint pressure in the lateral compartment [20]. Our radiological results are similar to those reported in previous studies and indicate that DFO is a very reliable method for correcting valgus malalignment as measured in long leg standing radiographs. The limb axis was consistently corrected to neutral with only one case of secondary loss of correction. However, as described above, the success of the operation regarding the radiological correction of the alignment does not necessarily correlate with a satisfying clinical outcome. The relative high incidence of fracture of the contralateral hinge intraoperatively in our study –slight lower than the literature- (in our study: MCDFO 27%, LODFO 33%; in the literature MCDFO 48%, LODFO 39% [29]) does not seem to cause a loss of correction postoperatively. Recent literature has suggested that fracture of the contralateral hinge increases the risk of delayed union or nonunion [14]. In our study, while statistical analysis was not possible, four of five cases presenting with delayed and nonunion had a fracture of the contralateral hinge.

### Complications and need for hardware removal

Generally, the reported incidence of complications in the available reviews ranged from 9% to 33% [7, 13, 31]. In our case series, we chose to report minor and major complications separately and found an incidence of major complications of 14% and of minor complications of 25%. This incidence of complications indicates that DFOs are far from risk free. Therefore, it is essential that the surgeon anticipates possible complications, closely follows the patient, initiates early treatment when needed, and informs the patient of the relative high chance of deviation from the normal postoperative course.

The mean time to bone union reported in the literature is around 4 months for MCDFO and 3–6 months for LCDFO [13]. Furthermore, the reported prevalence of delayed union is around 4% for MCDFO and around 6% for LCDFO [31]. However, there is a lack of a generally accepted definition for bone union, delayed and nonunion [30]. Above all, delayed union is mostly vaguely defined as failure of bone union in a reasonable time frame making comparison of different reports impossible. Interestingly, the available reviews on DFOs do not provide a definition of delayed union or nonunion. In our series we found a median bone union time of 6 months, an incidence of delayed union of 14% and an incidence of nonunion of 4%. Our results for nonunions are comparable to those of the literature, and the discrepancy in delayed union incidence might be due to the definition of bone union applied. In this study, we used clinical (lack of pain at the sight of the osteotomy) in addition to radiological criteria as definition of bone union. The use of clinical criteria is in our opinion appropriate since in MCDFOs commonly the osteotomy gap can hardly be recognized in radiographs directly postoperatively and therefore solely relying on “bony bridging” of the osteotomy to define union seems inappropriate.

The rate of hardware removal in our study was substantially higher than that reported in the literature (71% vs around 30%) [7, 13, 31]. This is interesting especially because most DFOs in our study were MCDFOs, which are considered to have a lower need for hardware removal since there is no friction between the plate and the iliotibial tract. A possible explanation for our high rate of hardware removal is the health care system in which the current study was performed that reimburses hardware removal operations even for minor hardware-related complaints.

### Survivorship: conversion to TKA/UKA

Survivorship of DFOs and the possibility to prevent or postpone the need of joint replacement is the major outcome of interest for surgeons and patients. The survival rate in our study was 93% at 59 months after the DFO. This is in accordance with the literature suggesting survival rates from 80% to 90% at 4–5 years after the operation [7, 13, 31]. This underlines that DFO is a successful operation for its main purpose, which is important because TKAs in young active patients has been shown to have poor survivorship and therefore the orthopaedic community is in need of a viable alternative for this patient population [4].

### Strengths and limitations

Strengths of this study include the clear indications for each technique and the consistent clinical and radiological documentation. The study is limited by the relatively small sample size, the retrospective design, the heterogeneity of the patients, and the lack of control group. Furthermore, there were no specific ROMs or clinical tests used. However, randomization between the MCDFO and LODFO is not possible because—in our opinion—each technique is appropriate for different patient groups. Furthermore, DFOs are not common even in specialized centers and it would be unrealistic to expect a controlled study with a large sample size on this topic in the near future.

## Conclusion

DFO is a reasonable treatment for lateral osteoarthritis in young patients to avoid disease progression and the need for an early UKA/TKA. However, there is a long rehabilitation time, a considerable risk for complications, and a high need for hardware removal. While a great part of the patients experienced symptoms at the long-term follow-up, most were satisfied with the result of the operation. Appropriate patient information is essential. Although statistical analysis identifying specific factors leading to complications was not possible in this study, it is worth noting that 80% of the cases with delayed and non-union had a fracture of the contralateral hinge.

## Data Availability

The data that support the findings of this study are available from the corresponding author, [PI], upon request.

## Abbreviations

DFO: Distal femoral osteotomy
MCDFO: Medial closing distal femoral osteotomy
LODFO: Lateral opening distal femoral osteotomy
BMI: Body mass index
HTO: High tibial osteotomy
AP: Anteroposterior
HKA: Hip knee ankle angle
MPTA: Mechanical medial proximal tibial angle
mLDFA: Mechanical lateral distal femoral angle
ADL: Activities of daily living
ROM: Range of motion
UKA: Unicompartmental arthroplasty
TKA: Total knee arthroplasty
K/L: Kellgren–Lawrence

## References

[1] Abdel Khalik H, Lameire DL, Rubinger L, Ekhtiari S, Khanna V, Ayeni OR (2022). Return to sport and work following distal femoral varus osteotomy: a systematic review. HSS J.

[2] Cerejo R, Dunlop DD, Cahue S, Channin D, Song J, Sharma L (2002). The influence of alignment on risk of knee osteoarthritis progression according to baseline stage of disease. Arthritis Rheum.

[3] Chang A, Hochberg M, Song J (2010). Frequency of varus and valgus thrust and factors associated with thrust presence in persons with or at higher risk of developing knee osteoarthritis. Arthritis Rheum.

[4] Charette RS, Sloan M, DeAngelis RD, Lee GC (2019). Higher rate of early revision following primary total knee arthroplasty in patients under age 55: a cautionary tale. J Arthroplasty.

[5] Cooke D, Scudamore A, Li J, Wyss U, Bryant T, Costigan P (1997). Axial lower-limb alignment: comparison of knee geometry in normal volunteers and osteoarthritis patients. Osteoarthritis Cartilage.

[6] Corrales LA, Morshed S, Bhandari M, Miclau T (2008). Variability in the assessment of fracture-healing in orthopaedic trauma studies. J Bone Joint Surg Am.

[7] Diaz CC, Lavoie-Gagne OZ, Knapik DM, Korrapati A, Chahla J, Forsythe B (2022). Outcomes of distal femoral osteotomy for valgus malalignment: a systematic review and meta-analysis of closing wedge versus opening wedge techniques. Am J Sports Med.

[8] Duethman NC, Bernard CD, Camp CL, Krych AJ, Stuart MJ (2019). Medial closing wedge distal femoral osteotomy. Clin Sports Med.

[9] Felson DT, Niu J, Gross KD (2013). Valgus malalignment is a risk factor for lateral knee osteoarthritis incidence and progression: findings from the multicenter osteoarthritis study and the osteoarthritis initiative. Arthritis Rheum.

[10] Fujita K, Sawaguchi T, Goshima K, Shigemoto K, Iwai S (2021). Influence of lateral hinge fractures on biplanar medial closing-wedge distal femoral osteotomy for valgus knee: a new classification of lateral hinge fracture. Arch Orthop Trauma Surg.

[11] Kellgren JH, Lawrence J (1963). The epidemiology of chronic rheumatism: atlas of standard radiographs of arthritis.

[12] Kim TW, Lee MC, Cho JH, Kim JS, Lee YS (2019). The ideal location of the lateral hinge in medial closing wedge osteotomy of the distal femur: analysis of soft tissue coverage and bone density. Am J Sports Med.

[13] Kim YC, Yang JH, Kim HJ (2018). Distal femoral varus osteotomy for valgus arthritis of the knees: systematic review of open versus closed wedge osteotomy. Knee Surg Relat Res.

[14] Matsushita T, Mori A, Watanabe S (2022). Analysis of bone union after medial closing wedge distal femoral osteotomy using a new radiographic scoring system. Arch Orthop Trauma Surg.

[15] Nha KW, Chang YS, Shon OJ (2019). Where is the target point to prevent cortical hinge fracture in medial closing-wedge distal femoral varus osteotomy?. J Knee Surg.

[16] Paley D, Pfeil J (2000). Principles of deformity correction around the knee. Orthopade.

[17] Pearson RG, Clement RG, Edwards KL, Scammell BE (2016). Do smokers have greater risk of delayed and non-union after fracture, osteotomy and arthrodesis? A systematic review with meta-analysis. BMJ Open.

[18] Petersen W, Forkel P (2013). Medial closing wedge osteotomy for correction of genu valgum and torsional malalignment. Oper Orthop Traumatol.

[19] Pilone C, Rosso F, Cottino U, Rossi R, Bonasia DE (2019). Lateral opening wedge distal femoral osteotomy for lateral compartment arthrosis/overload. Clin Sports Med.

[20] Quirno M, Campbell KA, Singh B (2017). Distal femoral varus osteotomy for unloading valgus knee malalignment: a biomechanical analysis. Knee Surg Sports Traumatol Arthrosc.

[21] Rensing N, Prabhakar G, Kusnezov N, Zarkadis NJ, Waterman BR, Pallis M (2019). Distal femoral osteotomy in a young symptomatic population: Outcomes correlate to concomitant pathology. J Orthop.

[22] Rosso F, Margheritini F (2014). Distal femoral osteotomy. Curr Rev Musculoskelet Med.

[23] Ryder CT, Crane L (1953). Measuring femoral anteversion: the problem and a method. J Bone Joint Surg Am.

[24] Sharma L, Song J, Felson DT, Cahue S, Shamiyeh E, Dunlop DD (2001). The role of knee alignment in disease progression and functional decline in knee osteoarthritis. JAMA.

[25] Sherman SL, Thompson SF, Clohisy JCF (2018). Distal femoral varus osteotomy for the management of valgus deformity of the knee. J Am Acad Orthop Surg.

[26] Sink EL, Leunig M, Zaltz I, Gilbert JC, Clohisy J, Academic Network for Conservational Hip Outcomes Research G (2012). Reliability of a complication classification system for orthopaedic surgery. Clin Orthop Relat Res.

[27] Staheli LT, Engel GM (1972). Tibial torsion: a method of assessment and a survey of normal children. Clin Orthop.

[28] Willey M, Wolf BR, Kocaglu B, Amendola A (2010). Complications associated with realignment osteotomy of the knee performed simultaneously with additional reconstructive procedures. Iowa Orthop J.

[29] Winkler PW, Rupp MC, Lutz PM (2021). A hinge position distal to the adductor tubercle minimizes the risk of hinge fractures in lateral open wedge distal femoral osteotomy. Knee Surg Sports Traumatol Arthrosc.

[30] Wittauer M, Burch MA, McNally M (2021). Definition of long-bone nonunion: a scoping review of prospective clinical trials to evaluate current practice. Injury.

[31] Wylie JD, Jones DL, Hartley MK (2016). Distal femoral osteotomy for the valgus knee: medial closing wedge versus lateral opening wedge: a systematic review. Arthroscopy.

